# Supplementary material for: Polymorphic Covalent Organic Frameworks: Molecularly Defined Pore Structures and Iodine Adsorption Property
Source: Molecules. 2023 Jan 3;28(1):449. doi: 10.3390/molecules28010449 (PMC9824140; doi:10.3390/molecules28010449)
Supplement: Supplementary file 1 [file molecules-28-00449-s001.zip › molecules-2058736-supplementary.pdf]

Supporting Information

Polymorphic covalent organic frameworks: molecularly defined pore structures and iodine adsorption property

Canran Wang[1], Shan Jiang[3], Wenyue Ma[1], Zhaoyang Liu[1,*] Leijing Liu[1], Yongcun Zou[2], Bin Xu[1], Wenjing Tian[1,*]

[1] State Key Laboratory of Supramolecular Structure and Materials, College of Chemistry，Jilin University, Changchun 130012, China

[2] State Key Laboratory of Inorganic Synthesis and Preparative Chemistry，College of Chemistry，Jilin University，Changchun 130012，China

[3] College of Chemistry and Chemical Engineering，Liaoning Normal University，Dalian 116029, China

* Correspondence: zhaoyangliu@jlu.edu.cn (Z.L.); wjtian@jlu.edu.cn (W.T.)

Contents

Section S1 Simulation of two-dimensional structures……….……….3

Section S2 Unit cell parameters………………………………………4

Section S3 SEM analysis………………………………………………36

Section S4 Thermogravimetric analysis and **structural** stability….……37

Section S5 Iodine solution adsorption experiment………………….…38

Section S6 Iodine vapor adsorption experiment……..……………………39

Section S1 Simulation of two-dimensional structures

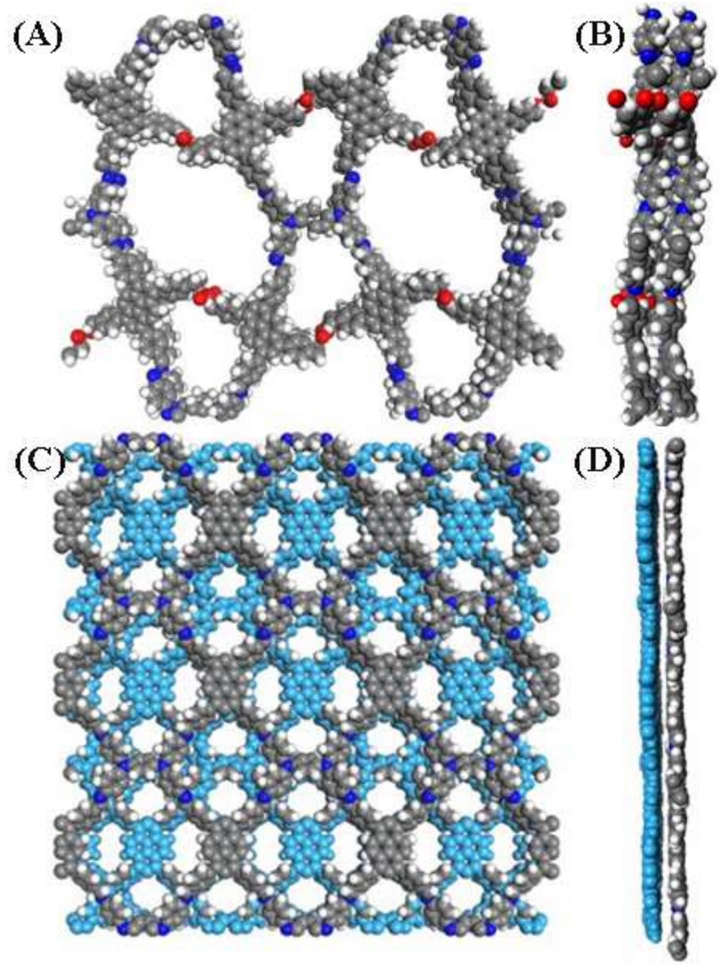

Figure S1. (A) Top view of the simulated two-dimensional AA stacking model structure of PyT-1; (B) Side view of the simulated

two-dimensional AA stacking model structure of PyT-1; (C) Top view of the simulated two-dimensional AB stacking model structure of PyT-2; (D) Side view of the simulated two-dimensional AB stacking model structure of PyT-2.

Section S2 Unit cell parameters

Pyt-1			
Triclinic P1			
a=34.8957 Å, b=56.3226 Å, c=9.2810 Å			
α = 89.7348°,β = 89.2027°,γ =92.3580°			
C1	0.51666	0.23691	1.17777
C2	0.50021	0.25941	1.1586
C3	0.46077	0.26067	1.12441
C4	0.43832	0.23943	1.11677
C5	0.45423	0.21754	1.13772
C6	0.49338	0.21579	1.16689
C7	0.55624	0.23561	1.20916
C8	0.57734	0.25683	1.23774
C9	0.56132	0.27886	1.219
C10	0.52309	0.28059	1.17591
C11	0.50993	0.19344	1.18389
C12	0.54951	0.19247	1.2015
C13	0.57324	0.21308	1.21385
C14	0.44465	0.28314	1.10382
C15	0.46795	0.30376	1.11865
C16	0.50661	0.30289	1.15527
C17	0.61558	0.21001	1.22169
C18	0.48691	0.17066	1.18529
C19	0.52821	0.32593	1.17202
C20	0.40355	0.28612	1.06944
C21	0.64153	0.22418	1.13775
C22	0.68066	0.21973	1.13514
C23	0.69447	0.20034	1.21105
C24	0.66889	0.18622	1.29559
C25	0.62995	0.19112	1.30178
C26	0.45691	0.16691	1.28586
C27	0.4352	0.14553	1.28882
C28	0.44338	0.12737	1.19178
C29	0.47399	0.13086	1.09279
C30	0.49573	0.15231	1.09015

C31	0.38662	0.27456	0.95134
C32	0.34792	0.27724	0.92076
C33	0.32565	0.29206	1.00597
C34	0.34275	0.30418	1.1218
C35	0.38138	0.30129	1.15295
C36	0.55988	0.33204	1.08268
C37	0.57781	0.3547	1.09016
C38	0.56416	0.37161	1.18677
C39	0.53279	0.36529	1.27775
C40	0.51494	0.3427	1.27003
C41	-0.27694	0.15102	1.23785
C42	0.41895	0.10536	1.19262
C43	0.58244	0.39574	1.19101
C44	0.28478	0.29506	0.9751
C45	0.16468	0.01771	0.94913
C46	0.13434	0.99614	1.88918
C47	0.17384	0.99304	1.89547
N48	0.12317	0.02035	0.95959
C49	0.05108	1.00029	1.93905
C50	0.10642	0.0435	0.96542
C51	0.0174	0.99929	1.85797
C52	-0.00668	0.97882	1.85903
C53	0.00243	0.95893	1.94004
C54	0.03514	0.96026	2.02729
C55	0.05845	0.98108	2.02998
C56	0.07541	0.04727	1.0595
C57	0.05972	0.0696	1.07031
C58	0.0749	0.08879	0.98967
C59	0.10496	0.08507	0.89077
C60	0.11974	0.06239	0.87601
N61	-0.02111	0.93763	1.92353
N62	0.05875	0.11149	1.01348
O63	0.13261	0.72625	1.28755
O64	0.65879	0.83416	1.70074

H65	0.40782	0.23939	1.0986
H66	0.43534	0.20194	1.12857
H67	0.60639	0.25665	1.27673
H68	0.57923	0.29456	1.23955
H69	0.56194	0.17517	1.20543
H70	0.45576	0.32091	1.1006
H71	0.63142	0.23812	1.06899
H72	0.70004	0.23093	1.06977
H73	0.67901	0.17134	1.35673
H74	0.61097	0.18005	1.36957
H75	0.4502	0.18073	1.3611
H76	0.41197	0.14318	1.36692
H77	0.48067	0.1171	1.01634
H78	0.51882	0.15485	1.01163
H79	0.40344	0.26329	0.88398
H80	0.3353	0.26807	0.82936
H81	0.32609	0.31567	1.18928
H82	0.39378	0.31044	1.24473
H83	0.57011	0.3194	1.00538
H84	0.60179	0.35919	1.01839
H85	0.52195	0.37792	1.35432
H86	0.49058	0.33825	1.34018
H87	-0.2653	0.13864	1.3137
H88	0.39778	0.10293	1.27805
H89	0.60618	0.40004	1.11817
H90	0.26786	0.30581	1.04567
H91	0.11625	0.98076	1.86437
H92	0.1834	0.97511	1.89012
H93	0.05657	0.01344	0.81951
H94	-0.03175	0.97796	1.79155
H95	0.04295	0.94554	2.09395
H96	0.0829	0.98208	2.10046
H97	0.06403	0.03302	1.12794
H98	0.03642	0.07231	1.14599

H99	0.1165	0.09925	0.82245
H100	0.14173	0.05954	0.79524
C101	-0.11599	0.79262	1.76468
C102	-0.09954	0.77233	1.70158
C103	-0.06011	0.77311	1.66066
C104	-0.03766	0.79376	1.68991
C105	-0.05356	0.81337	1.75347
C106	-0.09271	0.81334	1.79052
C107	-0.15556	0.79204	1.80306
C108	-0.17665	0.7703	1.79349
C109	-0.16064	0.75045	1.73176
C110	-0.12242	0.75123	1.68169
C111	-0.10925	0.83358	1.85128
C112	-0.14882	0.83342	1.87595
C113	-0.17255	0.8131	1.85254
C114	-0.044	0.75292	1.59631
C115	-0.0673	0.73248	1.57519
C116	-0.10595	0.73121	1.61756
C117	-0.21488	0.81543	1.87232
C118	-0.08622	0.85524	1.89194
C119	-0.12756	0.70837	1.59344
C120	-0.00291	0.75207	1.55172
C121	-0.24086	0.80629	1.76999
C122	-0.27998	0.81049	1.78194
C123	-0.29378	0.82484	1.89263
C124	-0.26817	0.83391	1.99549
C125	-0.22923	0.8291	1.98613
C126	-0.05619	0.85364	1.98982
C127	-0.03448	0.87386	2.02952
C128	-0.04267	0.89617	1.97285
C129	-0.07331	0.89793	1.87707
C130	-0.09505	0.87762	1.83754
C131	0.01399	0.76933	1.45876
C132	0.05269	0.76855	1.4187

C133	0.07497	0.75009	1.46823
C134	0.05789	0.73243	1.55802
C135	0.01928	0.73338	1.599
C136	-0.15925	0.70713	1.50202
C137	-0.17718	0.68516	1.46924
C138	-0.16352	0.66409	1.527
C139	-0.13212	0.66543	1.6204
C140	-0.11426	0.68736	1.65285
C141	0.67764	0.87431	1.86061
C142	-0.01825	0.91712	2.0113
C143	-0.18179	0.64088	1.48844
C144	0.11583	0.74904	1.42695
C145	0.23599	1.01418	1.90585
C146	0.26633	0.03613	0.95153
C147	0.22683	0.03857	0.96945
N148	0.2775	1.0113	1.90438
C149	0.3496	0.02991	0.97818
C150	0.29425	0.98909	1.86375
C151	0.38326	0.03527	0.8979
C152	0.40734	0.05474	0.93371
C153	0.39825	0.06933	1.04945
C154	0.36557	0.06334	1.13483
C155	0.34225	0.04334	1.1018
C156	0.32527	0.98069	1.94115
C157	0.34096	0.95899	1.90695
C158	0.32576	0.94495	1.79661
C159	0.29568	0.95356	1.7144
C160	0.2809	0.9758	1.74538
N161	0.4218	0.09052	1.07024
N162	0.34191	0.92222	1.77399
O163	0.26798	0.32395	0.88827
O164	-0.25812	0.1976	1.15874
H165	-0.00716	0.79488	1.66803
H166	-0.03467	0.82873	1.77122

H167	-0.20569	0.76831	1.83535
H168	-0.17856	0.73439	1.72449
H169	-0.16124	0.84958	1.91417
H170	-0.05512	0.71719	1.52387
H171	-0.23077	0.7967	1.6769
H172	-0.29938	0.80321	1.7018
H173	-0.27827	0.84478	2.08312
H174	-0.21024	0.83611	2.06837
H175	-0.04947	0.83659	2.03418
H176	-0.01123	0.87209	2.10449
H177	-0.08001	0.91498	1.83142
H178	-0.11816	0.87923	1.76179
H179	-0.00284	0.78349	1.41863
H180	0.06528	0.78211	1.34723
H181	0.07456	0.71805	1.59785
H182	0.0069	0.71981	1.6708
H183	-0.1695	0.72315	1.45403
H184	-0.20118	0.68457	1.39638
H185	-0.12127	0.64946	1.66763
H186	-0.08989	0.688	1.72403
H187	0.66601	0.88202	1.95772
H188	0.00295	0.91502	2.0937
H189	-0.20556	0.64053	1.41487
H190	0.13277	0.73519	1.47108
H191	0.28441	0.05211	0.95416
H192	0.21727	0.05584	0.99961
H193	0.34407	1.02549	1.77411
H194	0.4324	0.05921	0.8674
H195	0.35777	0.07378	1.22702
H196	0.31782	0.03858	1.17059
H197	0.33667	0.99066	2.03117
H198	0.36427	0.95254	1.97007
H199	0.28411	0.94363	1.62463
H200	0.25889	0.98265	1.67747

C201	0.00692	0.29658	1.27586
C202	-0.00751	0.27498	1.21393
C203	-0.04678	0.27239	1.17602
C204	-0.07106	0.29105	1.20705
C205	-0.05712	0.31193	1.26947
C206	-0.01819	0.31523	1.30354
C207	0.04631	0.29937	1.31122
C208	0.06929	0.27952	1.29998
C209	0.05525	0.25838	1.23939
C210	0.01722	0.25591	1.19223
C211	-0.00366	0.33681	1.36312
C212	0.03573	0.34001	1.38478
C213	0.06124	0.3218	1.35949
C214	-0.06088	0.25091	1.11282
C215	-0.03579	0.23254	1.08985
C216	0.00274	0.23456	1.12928
C217	0.10316	0.32773	1.37606
C218	-0.02863	0.35641	1.40561
C219	0.02637	0.21366	1.10343
C220	-0.10164	0.24657	1.07135
C221	0.13009	0.32084	1.27171
C222	0.16866	0.32836	1.28069
C223	0.18088	0.34383	1.39039
C224	0.15433	0.35066	1.49525
C225	0.11599	0.34255	1.48883
C226	-0.05862	0.35225	1.50578
C227	-0.08214	0.37054	1.5472
C228	-0.07586	0.39346	1.48998
C229	-0.04528	0.39784	1.39186
C230	-0.02172	0.37946	1.35061
C231	-0.11982	0.26232	0.97973
C232	-0.15822	0.25826	0.94261
C233	-0.17887	0.23797	0.99378
C234	-0.16047	0.22182	1.08221

C235	-0.12217	0.22605	1.12026
C236	0.05825	0.21513	1.00959
C237	0.07815	0.19478	0.97537
C238	0.0663	0.17262	1.0341
C239	0.0347	0.17127	1.1299
C240	0.01488	0.19159	1.16379
C241	0.22076	0.35351	1.3939
C242	-0.10216	0.41225	1.53037
C243	0.08668	0.15106	0.99406
C244	-0.2194	0.23344	0.9556
C245	0.64812	0.53049	1.40599
C246	0.66165	0.5543	1.42059
C247	0.70074	0.5601	1.43551
N248	0.60702	0.52408	1.40768
C249	0.57919	0.54101	1.45356
C250	0.59242	0.50053	1.36824
C251	0.54535	0.54348	1.37585
C252	0.51954	0.56082	1.41356
C253	0.52703	0.57611	1.52867
C254	0.55994	0.57293	1.61156
C255	0.58504	0.555	1.57668
C256	0.56209	0.48952	1.44798
C257	0.54848	0.46657	1.4149
C258	0.56513	0.45388	1.30335
C259	0.5945	0.46503	1.21887
C260	0.60716	0.48845	1.2488
N261	0.50163	0.59521	1.55133
N262	0.55112	0.42986	1.28187
O263	0.63435	0.84088	1.35843
O264	0.15718	0.71662	1.62704
H265	-0.10149	0.28956	1.18749
H266	-0.07735	0.32562	1.28871
H267	0.09831	0.28	1.33962
H268	0.07456	0.24391	1.2307

H269	0.04657	0.35717	1.42211
H270	-0.04644	0.21627	1.0394
H271	0.12109	0.31044	1.17935
H272	0.18881	0.32277	1.19905
H273	0.16322	0.36234	1.58215
H274	0.09625	0.34792	1.57254
H275	-0.06388	0.33469	1.5506
H276	-0.10531	0.36678	1.62394
H277	-0.04004	0.4154	1.34576
H278	0.00132	0.38305	1.2731
H279	-0.10424	0.27787	0.93836
H280	-0.17182	0.27069	0.87214
H281	-0.17587	0.20606	1.12327
H282	-0.10878	0.21359	1.19108
H283	0.06713	0.23197	0.96087
H284	0.10227	0.19624	0.90068
H285	0.02522	0.15443	1.17791
H286	-0.00962	0.19014	1.23684
H287	0.23144	0.36217	1.49016
H288	-0.12326	0.40835	1.61438
H289	0.11054	0.15274	0.91868
H290	-0.23512	0.2182	1.00098
H291	0.64219	0.56868	1.42465
H292	-0.30337	0.53489	1.5036
H293	0.53971	0.53256	1.28251
H294	0.49431	0.56314	1.34917
H295	0.56657	0.58398	1.70319
H296	0.60965	0.55233	1.6436
H297	0.54965	0.49847	1.53892
H298	0.5257	0.45815	1.47978
H299	0.60711	0.45614	1.12818
H300	0.62862	0.49716	1.17923
C301	0.39376	0.73295	1.66659
C302	0.40818	0.75677	1.64624

C303	0.44744	0.76138	1.60905
C304	0.47172	0.74214	1.59963
C305	0.45779	0.71898	1.62172
C306	0.41887	0.71389	1.65387
C307	0.35438	0.72827	1.701
C308	0.3314	0.74762	1.73126
C309	0.34542	0.77092	1.71137
C310	0.38345	0.77591	1.66537
C311	0.40435	0.69022	1.67205
C312	0.36496	0.68587	1.69267
C313	0.33945	0.70439	1.7069
C314	0.46152	0.78515	1.58731
C315	0.43643	0.8037	1.60398
C316	0.39791	0.79954	1.64355
C317	0.29753	0.69771	1.71796
C318	0.42933	0.66949	1.67162
C319	0.37429	0.82064	1.66203
C320	0.50228	0.79162	1.54981
C321	0.27059	0.70962	1.63604
C322	0.23201	0.70185	1.63638
C323	0.21982	0.68136	1.71328
C324	0.24639	0.66947	1.79583
C325	0.28473	0.67766	1.79908
C326	0.45933	0.6683	1.76991
C327	0.48286	0.64885	1.77113
C328	0.47656	0.63007	1.67465
C329	0.44596	0.63095	1.578
C330	0.42239	0.65046	1.57708
C331	0.52043	0.78156	1.43037
C332	0.55882	0.78753	1.39685
C333	0.57949	0.80419	1.48043
C334	0.56111	0.81479	1.59761
C335	0.52283	0.80862	1.63169
C336	0.34238	0.82404	1.57511

C337	0.32248	0.84508	1.58403
C338	0.33434	0.86308	1.67966
C339	0.36597	0.85945	1.76824
C340	0.3858	0.83847	1.75909
C341	0.17994	0.67181	1.70456
C342	0.50286	0.61023	1.67355
C343	0.31397	0.88556	1.68539
C344	0.62001	0.81066	1.44645
C345	-0.24745	0.5014	1.44899
C346	-0.26098	0.47798	1.42012
C347	-0.30007	0.47152	1.42941
N348	-0.20636	0.50756	1.45629
C349	-0.17851	0.4892	1.46367
C350	-0.19176	0.53205	1.46092
C351	-0.1447	0.49108	1.38001
C352	-0.11888	0.47274	1.37918
C353	-0.12635	0.45215	1.46082
C354	-0.15924	0.45067	1.55057
C355	-0.18434	0.46943	1.55511
C356	-0.16141	0.53844	1.55266
C357	-0.1478	0.56202	1.56236
C358	-0.16447	0.57986	1.48293
C359	-0.19387	0.57359	1.3863
C360	-0.20653	0.54974	1.37259
N361	-0.10094	0.43293	1.44244
N362	-0.15046	0.60384	1.50559
O363	-0.23376	0.20932	0.81739
O364	0.24348	0.31514	1.23244
H365	0.50215	0.7447	1.57914
H366	0.47802	0.70505	1.61108
H367	0.30239	0.74495	1.77246
H368	0.32612	0.78503	1.73334
H369	0.35413	0.66758	1.69749
H370	0.44708	0.82183	1.58506

H371	0.27956	0.72438	1.56654
H372	0.21184	0.71137	1.57252
H373	0.23751	0.65377	1.85771
H374	0.30448	0.66825	1.86539
H375	0.46461	0.68263	1.84468
H376	0.50605	0.64848	1.84747
H377	0.44071	0.61668	1.50199
H378	0.39933	0.65104	1.50032
H379	0.50484	0.76891	1.36425
H380	0.57241	0.77949	1.30445
H381	0.57653	0.82765	1.66386
H382	0.50945	0.81667	1.72445
H383	0.33349	0.81058	1.49854
H384	0.29834	0.84751	1.5141
H385	0.37547	0.87295	1.84404
H386	0.41031	0.83611	1.82737
H387	0.16928	0.65848	1.78126
H388	0.52399	0.60961	1.75737
H389	0.29008	0.88783	1.61436
H390	0.63574	0.82281	1.51578
H391	-0.24153	0.46419	1.39388
H392	0.70404	0.49606	1.38613
H393	-0.13908	0.50638	1.31111
H394	-0.09367	0.47403	1.30978
H395	-0.16585	0.43534	1.61777
H396	-0.20894	0.46834	1.62746
H397	-0.14895	0.52521	1.6202
H398	-0.125	0.5667	1.63628
H399	-0.20649	0.58675	1.3189
H400	-0.228	0.54504	1.29347
C401	0.80929	0.2333	0.97448
C402	0.79385	0.25529	0.93301
C403	0.75448	0.25623	0.89703
C404	0.73111	0.23539	0.90977

C405	0.74603	0.21407	0.95238
C406	0.78509	0.2125	0.98391
C407	0.84879	0.23223	1.00777
C408	0.87083	0.25364	1.01564
C409	0.8558	0.27516	0.97508
C410	0.81766	0.27632	0.92987
C411	0.80063	0.19061	1.02307
C412	0.84013	0.18949	1.04244
C413	0.86475	0.20983	1.03498
C414	0.73937	0.27815	0.8542
C415	0.76358	0.29855	0.84917
C416	0.80218	0.29807	0.88716
C417	0.9069	0.20633	1.04687
C418	0.77662	0.16849	1.04625
C419	0.82481	0.32095	0.8816
C420	0.69843	0.28082	0.81628
C421	0.93336	0.2177	0.95086
C422	0.97223	0.21261	0.95369
C423	0.98524	0.19526	1.04797
C424	0.95915	0.18396	1.14453
C425	0.9205	0.18957	1.14479
C426	0.7466	0.16793	1.14831
C427	0.72398	0.14722	1.17166
C428	0.73122	0.12656	1.09405
C429	0.76184	0.12688	0.99383
C430	0.7845	0.14768	0.97071
C431	0.68088	0.26651	0.71074
C432	0.64232	0.26895	0.67693
C433	0.62085	0.28622	0.74579
C434	0.63859	0.30101	0.84855
C435	0.67706	0.29839	0.88312
C436	0.85661	0.32411	0.78833
C437	0.87553	0.34642	0.7739
C438	0.86277	0.36592	0.85218

C439	0.83126	0.3626	0.94727
C440	0.81241	0.34034	0.9614
C441	0.01308	0.16243	1.09711
C442	0.70584	0.10521	1.11587
C443	0.8821	0.3896	0.83313
C444	0.58013	0.289	0.71131
C445	0.44775	0.01607	0.95521
C446	0.46234	1.00864	0.91935
C447	0.50165	1.00512	0.92964
N448	0.40643	0.0196	0.96183
C449	0.37945	1.01537	0.96223
C450	0.39075	0.0429	0.94437
C451	0.34568	1.01274	0.88238
C452	0.32073	0.99292	0.90291
C453	0.32902	0.97528	1.00258
C454	0.36183	0.9784	1.08818
C455	0.38605	0.99869	1.07098
C456	0.36006	0.04962	1.0326
C457	0.3454	0.07222	1.02089
C458	0.36134	0.08881	0.92291
C459	0.39108	0.08202	0.82985
C460	0.4048	0.05898	0.83799
N461	0.30454	0.95415	1.00662
N462	0.34625	0.11213	0.92364
O463	0.44786	0.72588	0.59142
O464	0.97855	0.83531	0.90671
H465	0.70063	0.23534	0.89109
H466	0.72647	0.1987	0.95819
H467	0.89988	0.25404	1.05504
H468	0.87442	0.29094	0.98038
H469	0.85176	0.17231	1.06366
H470	0.75216	0.31522	0.81418
H471	0.92382	0.2298	0.86907
H472	0.99201	0.22164	0.87873

H473	0.96865	0.17072	1.21972
H474	0.90112	0.18074	1.222
H475	0.74061	0.1837	1.20872
H476	0.70076	0.14734	1.25037
H477	0.7678	0.11115	0.93214
H478	0.80758	0.14774	0.8914
H479	0.69709	0.25332	0.65585
H480	0.62922	0.25765	0.59548
H481	0.62255	0.31443	0.90334
H482	0.68996	0.30968	0.96495
H483	0.86617	0.3094	0.72481
H484	0.8996	0.34857	0.69937
H485	0.82109	0.37729	1.01007
H486	0.78797	0.3382	1.03429
H487	0.02423	0.15203	1.18439
H488	0.68468	0.10541	1.20194
H489	0.90593	0.39154	0.7577
H490	0.5638	0.30177	0.76988
H491	0.44357	0.99305	0.90954
H492	0.51039	0.9871	0.94225
H493	0.33947	0.01009	0.82872
H494	0.29558	0.99066	0.83655
H495	0.36905	0.96551	1.16858
H496	0.41058	1.00118	1.14014
H497	0.34814	0.03754	1.1138
H498	0.32235	0.07728	1.09223
H499	0.40317	0.09404	0.74884
H500	0.42654	0.05367	0.76173
C501	0.20313	0.80808	0.99005
C502	0.21858	0.78609	0.9482
C503	0.25794	0.78515	0.90815
C504	0.28131	0.80599	0.91728
C505	0.26639	0.82731	0.96025
C506	0.22733	0.82888	0.99577

C507	0.16363	0.80915	1.02741
C508	0.14159	0.78774	1.03879
C509	0.15662	0.76622	0.99786
C510	0.19476	0.76506	0.94873
C511	0.21179	0.85077	1.03534
C512	0.17229	0.85189	1.05878
C513	0.14767	0.83155	1.05504
C514	0.27305	0.76323	0.86497
C515	0.24884	0.74283	0.86362
C516	0.21024	0.74331	0.90562
C517	0.10552	0.83504	1.07113
C518	0.2358	0.87289	1.05476
C519	0.18761	0.72043	0.90372
C520	0.31399	0.76056	0.82292
C521	0.07906	0.82368	0.97853
C522	0.0402	0.82877	0.98514
C523	0.02719	0.84612	1.07981
C524	0.05327	0.85742	1.17301
C525	0.09193	0.85181	1.16954
C526	0.26582	0.87345	1.15364
C527	0.28845	0.89416	1.17347
C528	0.2812	0.91482	1.09545
C529	0.25059	0.91449	0.99845
C530	0.22792	0.8937	0.97888
C531	0.33154	0.77487	0.71474
C532	0.3701	0.77243	0.67703
C533	0.39158	0.75516	0.74461
C534	0.37383	0.74037	0.85006
C535	0.33536	0.74299	0.88851
C536	0.15581	0.71727	0.81396
C537	0.13689	0.69495	0.80276
C538	0.14965	0.67546	0.8808
C539	0.18116	0.67878	0.9724
C540	0.20001	0.70104	0.98331

C541	0.99934	0.87895	1.02539
C542	0.30658	0.93617	1.11342
C543	0.13032	0.65178	0.8651
C544	0.43229	0.75238	0.70603
C545	0.56467	1.02531	0.92075
C546	0.55008	0.03274	0.94217
C547	0.51077	0.03626	0.95638
N548	0.60599	1.02178	0.92324
C549	0.63298	0.02601	0.97676
C550	0.62167	0.99848	0.90545
C551	0.66674	0.02864	0.89322
C552	0.69169	0.04846	0.91003
C553	0.6834	0.0661	1.00958
C554	0.65059	0.06298	1.09878
C555	0.62637	0.04269	1.08526
C556	0.65236	0.99176	0.99085
C557	0.66702	0.96916	0.97888
C558	0.65108	0.95257	0.88349
C559	0.62134	0.95936	0.79316
C560	0.60762	0.9824	0.80144
N561	0.70789	0.08723	1.00987
N562	0.66617	0.92925	0.88396
O563	0.56456	0.3155	0.59684
O564	0.03387	0.20607	0.97381
H565	0.31179	0.80604	0.89541
H566	0.28595	0.84268	0.96315
H567	0.11254	0.78734	1.08125
H568	0.138	0.75044	1.00601
H569	0.16066	0.86907	1.08024
H570	0.26027	0.72616	0.82837
H571	0.08861	0.81157	0.89643
H572	0.02041	0.81974	0.91277
H573	0.04377	0.87066	1.24845
H574	0.1113	0.86064	1.24423

H575	0.27181	0.85768	1.21432
H576	0.31166	0.89404	1.24976
H577	0.24462	0.93023	0.93651
H578	0.20484	0.89364	0.90198
H579	0.31533	0.78806	0.6608
H580	0.3832	0.78373	0.59357
H581	0.38987	0.72695	0.90392
H582	0.32247	0.7317	0.97232
H583	0.14625	0.73198	0.75061
H584	0.11282	0.69281	0.73086
H585	0.19134	0.66409	1.03496
H586	0.22445	0.70318	1.05352
H587	0.98819	0.88935	1.11325
H588	0.32774	0.93597	1.19728
H589	0.10649	0.64984	0.79227
H590	0.44862	0.7396	0.76361
H591	0.56886	0.04833	0.92952
H592	0.50203	0.05427	0.96889
H593	0.67296	1.03129	0.78259
H594	0.71685	0.05072	0.84091
H595	0.64337	0.07587	1.17922
H596	0.60184	0.0402	1.15712
H597	0.66428	1.00384	1.07012
H598	0.69007	0.9641	1.04809
H599	0.60926	0.94734	0.71409
H600	0.58588	0.98771	0.72716
C601	0.30308	0.30286	0.99901
C602	0.28764	0.28009	0.9588
C603	0.24827	0.27714	0.92292
C604	0.2249	0.29678	0.93453
C605	0.23982	0.31887	0.97592
C606	0.27888	0.32243	1.0073
C607	0.34258	0.30595	1.03218
C608	0.36462	0.28567	1.04122

C609	0.34959	0.26338	1.00189
C610	0.31145	0.26028	0.95681
C611	0.29442	0.34511	1.04522
C612	0.33392	0.34825	1.06447
C613	0.35854	0.32917	1.05812
C614	0.23316	0.25444	0.88135
C615	0.25737	0.23528	0.87743
C616	0.29597	0.23773	0.91533
C617	0.40069	0.33482	1.06974
C618	0.27041	0.36601	1.06718
C619	0.3186	0.21601	0.91103
C620	0.19222	0.24969	0.84364
C621	0.42715	0.32481	0.97433
C622	0.46602	0.33187	0.97682
C623	0.47903	0.34989	1.07011
C624	0.45294	0.35986	1.16607
C625	0.41428	0.35227	1.16671
C626	0.24039	0.36504	1.16926
C627	0.21777	0.38459	1.19148
C628	0.22501	0.40562	1.11269
C629	0.25563	0.40686	1.01245
C630	0.27829	0.38722	0.99047
C631	0.17467	0.2631	0.73732
C632	0.13611	0.25869	0.7037
C633	0.11463	0.24033	0.77356
C634	0.13238	0.22644	0.87713
C635	0.17085	0.23103	0.9115
C636	0.3504	0.21447	0.8179
C637	0.36932	0.19312	0.80469
C638	0.35656	0.17297	0.88408
C639	0.32505	0.17469	0.97903
C640	0.3062	0.19598	0.99194
C641	0.51929	0.35893	1.06511
C642	0.19963	0.42568	1.13335

C643	0.37589	0.15028	0.86633
C644	0.07392	0.23546	0.7393
C645	0.95396	0.52749	0.91436
C646	0.96855	0.55119	0.90571
C647	1.00786	0.55671	0.91575
N648	0.91264	0.52184	0.92124
C649	0.88566	0.54023	0.94909
C650	0.89696	0.49775	0.90511
C651	0.85189	0.54113	0.86914
C652	0.82694	0.55968	0.88859
C653	0.83523	0.57774	0.98726
C654	0.86804	0.57629	1.07299
C655	0.89226	0.55724	1.0569
C656	0.86627	0.48946	0.99377
C657	0.85161	0.4661	0.98335
C658	0.86755	0.45033	0.88627
C659	0.89729	0.45864	0.79279
C660	0.91101	0.48238	0.79961
N661	0.81075	0.59762	0.99015
N662	0.85246	0.42624	0.88834
O663	0.95407	0.83321	0.56192
O664	0.47234	0.72503	0.93629
H665	0.19442	0.29527	0.91589
H666	0.22026	0.33323	0.98089
H667	0.39367	0.28675	1.08061
H668	0.36821	0.24855	1.00806
H669	0.34555	0.36603	1.0847
H670	0.24594	0.21803	0.84339
H671	0.4176	0.31221	0.89324
H672	0.4858	0.32386	0.90235
H673	0.46244	0.37358	1.24051
H674	0.39491	0.36011	1.24345
H675	0.2344	0.34896	1.23057
H676	0.19455	0.38329	1.27023

H677	0.26159	0.4229	0.94987
H678	0.30137	0.38834	0.91112
H679	0.19088	0.27712	0.68167
H680	0.12301	0.26932	0.62163
H681	0.11634	0.2122	0.93269
H682	0.18374	0.22039	0.99394
H683	0.35996	0.22967	0.75354
H684	0.39339	0.1922	0.73024
H685	0.31488	0.15948	1.04267
H686	0.28176	0.19688	1.06475
H687	0.53044	0.36989	1.15179
H688	0.17847	0.42439	1.21946
H689	0.39972	0.14956	0.79098
H690	0.05759	0.22186	0.79861
H691	0.94978	0.56582	0.89505
H692	0.00418	0.54933	0.98104
H693	0.84568	0.52794	0.78769
H694	0.80179	0.56065	0.82215
H695	0.87526	0.58955	1.15266
H696	0.91679	0.55601	1.12616
H697	0.85435	0.50093	1.0743
H698	0.82856	0.45987	1.055
H699	0.90938	0.44724	0.71244
H700	0.93275	0.4888	0.72302
C701	0.70934	0.73852	0.96552
C702	0.72479	0.76129	0.92242
C703	0.76415	0.76424	0.88225
C704	0.78752	0.74459	0.89252
C705	0.7726	0.72251	0.93671
C706	0.73354	0.71895	0.97238
C707	0.66984	0.73543	1.003
C708	0.6478	0.75571	1.01321
C709	0.66283	0.778	0.97105
C710	0.70097	0.7811	0.9218

C711	0.718	0.69626	1.01319
C712	0.67851	0.69313	1.03675
C713	0.65388	0.71221	1.03191
C714	0.77926	0.78694	0.83782
C715	0.75505	0.8061	0.83536
C716	0.71645	0.80365	0.87744
C717	0.61173	0.70656	1.04826
C718	0.74201	0.67537	1.03382
C719	0.69382	0.82537	0.87428
C720	0.8202	0.79169	0.79556
C721	0.58527	0.71657	0.95506
C722	0.54641	0.70951	0.96201
C723	0.5334	0.69149	1.05767
C724	0.55948	0.68152	1.15146
C725	0.59814	0.68911	1.14763
C726	0.77203	0.67634	1.1327
C727	0.79466	0.65679	1.15365
C728	0.78741	0.63576	1.0768
C729	0.7568	0.63452	0.97983
C730	0.73413	0.65416	0.95912
C731	0.83775	0.77828	0.68816
C732	0.87631	0.78269	0.65026
C733	0.89779	0.80105	0.71684
C734	0.88004	0.81494	0.82148
C735	0.84157	0.81035	0.86014
C736	0.66202	0.82691	0.78439
C737	0.6431	0.84826	0.77197
C738	0.65586	0.86841	0.84889
C739	0.68737	0.86669	0.94064
C740	0.70622	0.84539	0.95277
C741	0.49313	0.68245	1.05739
C742	0.81279	0.6157	1.09593
C743	0.63653	0.8911	0.83189
C744	0.9385	0.80592	0.67804

C745	0.05846	0.51389	0.9616
C746	0.04387	0.49019	0.9558
C747	0.00456	0.48467	0.97027
N748	0.09978	0.51954	0.96383
C749	0.12676	0.50115	0.9899
C750	0.11546	0.54363	0.94471
C751	0.16053	0.50025	0.90646
C752	0.18548	0.4817	0.92434
C753	0.17719	0.46364	1.02489
C754	0.14438	0.46509	1.11397
C755	0.12016	0.48414	1.09934
C756	0.14615	0.55192	1.02969
C757	0.16081	0.57527	1.01642
C758	0.14487	0.59105	0.92013
C759	0.11513	0.58274	0.83022
C760	0.10141	0.559	0.83982
N761	0.20167	0.44376	1.02634
N762	0.15996	0.61514	0.91926
O763	0.05835	0.20817	0.62634
O764	0.54008	0.31635	0.94422
H765	0.818	0.74611	0.87061
H766	0.79216	0.70815	0.94044
H767	0.61875	0.75463	1.05569
H768	0.64421	0.79283	0.97833
H769	0.66687	0.67535	1.0592
H770	0.76648	0.82335	0.79916
H771	0.59482	0.72917	0.87226
H772	0.52662	0.71752	0.88915
H773	0.54998	0.6678	1.22767
H774	0.61751	0.68127	1.22278
H775	0.77802	0.69242	1.19248
H776	0.81787	0.65809	1.2299
H777	0.75083	0.61848	0.91878
H778	0.71105	0.65304	0.88225

H779	0.82154	0.76426	0.63499
H780	0.88941	0.77206	0.56742
H781	0.89608	0.82918	0.87457
H782	0.82868	0.82099	0.94333
H783	0.65246	0.81171	0.72189
H784	0.61903	0.84917	0.69999
H785	0.69755	0.8819	1.00236
H786	0.73066	0.8445	1.02307
H787	0.48198	0.67148	1.14585
H788	0.83395	0.61699	1.17976
H789	0.6127	0.89182	0.75899
H790	0.95483	0.81952	0.73488
H791	0.06265	0.47556	0.944
H792	1.00824	0.49205	0.93011
H793	0.16675	0.51344	0.82362
H794	0.21063	0.48073	0.85532
H795	0.13716	0.45182	1.19514
H796	0.09563	0.48537	1.1711
H797	0.15807	0.54045	1.10962
H798	0.18386	0.58151	1.08532
H799	0.10305	0.59414	0.7505
H800	0.07967	0.55258	0.76586
H794	0.21063	0.48073	0.85532
H795	0.13716	0.45182	1.19514
H796	0.09563	0.48537	1.1711
H797	0.15807	0.54045	1.10962
H798	0.18386	0.58151	1.08532
H799	0.10305	0.59414	0.7505
H800	0.07967	0.55258	0.76586

Table S1. Unit cell parameters of PyT-1 with AB stacking structure.

Pyt-2			
Triclinic P1			
a=7.6860 Å, b=21.6648 Å, 24.5280 Å			
α = 89.5052°,β = 88.1686°,γ =91.8171°			
C1	0.05174	0.94848	0.55905
C2	0.12252	0.89903	0.53062
C3	0.09137	0.94923	0.38598
C4	0.38255	0.0988	0.34645
C5	0.32974	0.15688	0.36482
C6	0.2719	0.20128	0.3281
C7	0.2665	0.18817	0.2724
C8	0.31846	0.1305	0.25396
C9	0.37522	0.08665	0.2904
C10	0.20996	0.23422	0.23289
C11	0.06492	0.53707	0.04284
C12	0.39092	0.6648	0.95437
C13	0.30751	0.72115	0.96049
C14	0.26571	0.75666	0.91564
C15	0.30445	0.73666	0.86302
C16	0.38667	0.68029	0.85546
C17	0.43043	0.6449	0.90095
N18	0.25776	0.77544	0.81884
C19	0.47084	0.56435	0.00071
N20	0.43803	0.62995	0.00255
C21	0.40879	0.04914	0.5567
C22	0.34887	0.09848	0.52621
C23	0.44723	0.04962	0.38335
C24	0.16118	0.89819	0.35232
C25	0.1373	0.83573	0.36761
C26	0.18941	0.78885	0.33211
C27	0.26631	0.804	0.28103
C28	0.29159	0.86614	0.26599
C29	0.23915	0.91252	0.30109
C30	0.30675	0.75569	0.24136

C31	0.09647	0.47401	0.04203
C32	0.07042	0.33976	0.94547
C33	0.47961	0.279	0.94278
C34	0.49272	0.2448	0.89514
C35	0.0976	0.27093	0.84884
C36	0.16789	0.3315	0.85035
C37	0.15427	0.36566	0.89847
N38	0.1311	0.23074	0.80412
C39	0.00648	0.0001	0.47105
C40	0.03364	0.00042	0.35783
C41	0.43433	0.04959	0.4411
C42	0.36163	0.09868	0.46955
C43	0.39471	0.04865	0.61417
C44	0.1016	0.899	0.65373
C45	0.15215	0.84065	0.63538
C46	0.20826	0.79595	0.67212
C47	0.21416	0.80905	0.72781
C48	0.16444	0.86698	0.74623
C49	0.10939	0.91112	0.70978
C50	0.2689	0.76271	0.76735
C51	0.44116	0.46422	0.95709
C52	0.12288	0.33684	0.04548
C53	0.20848	0.28092	0.03934
C54	0.25166	0.24564	0.08416
C55	0.21214	0.26544	0.1368
C56	0.12773	0.32138	0.14438
C57	0.0826	0.35654	0.09891
N58	0.26033	0.22691	0.18096
C59	0.03908	0.43686	0.99918
N60	0.07441	0.37144	0.99732
C61	0.07728	0.94881	0.44346
C62	0.13527	0.89917	0.47396
C63	0.03884	0.94851	0.6168
C64	0.32292	0.09933	0.64786

C65	0.34441	0.16187	0.6326
C66	0.29048	0.20847	0.66811
C67	0.21414	0.19296	0.71919
C68	0.19123	0.13073	0.73421
C69	0.24547	0.08462	0.69909
C70	0.17183	0.24104	0.75888
C71	0.41205	0.52746	0.95787
C72	0.44324	0.66163	0.05438
C73	0.01935	0.72201	0.05723
C74	0.00491	0.75613	0.10488
C75	0.41872	0.73061	0.15098
C76	0.34614	0.67036	0.14949
C77	0.35844	0.63612	0.10139
N78	0.38678	0.77097	0.19569
C79	0.48158	0.99821	0.52908
C80	0.45443	0.99777	0.6423
C81	0.5418	0.9471	0.55916
C82	0.6145	0.89802	0.53071
C83	0.58141	0.94805	0.38609
C84	0.8984	0.101	0.34627
C85	0.84785	0.15935	0.36462
C86	0.79174	0.20405	0.32788
C87	0.78584	0.19095	0.27219
C88	0.83556	0.13302	0.25377
C89	0.89061	0.08888	0.29022
C90	0.7311	0.23729	0.23265
C91	0.55884	0.53578	0.04291
C92	0.89199	0.66521	0.95436
C93	0.80638	0.72113	0.9605
C94	0.76321	0.75642	0.91567
C95	0.80273	0.73662	0.86304
C96	0.88714	0.68067	0.85545
C97	0.93227	0.64552	0.90093
N98	0.75454	0.77515	0.81887

C99	0.97579	0.5652	0.00066
N100	0.94046	0.63061	0.00252
C101	0.92272	0.05119	0.55654
C102	0.86473	0.10083	0.52604
C103	0.96116	0.05149	0.3832
C104	0.65321	0.89737	0.3524
C105	0.63171	0.83483	0.36767
C106	0.68565	0.78822	0.33215
C107	0.76199	0.80373	0.28107
C108	0.7849	0.86597	0.26606
C109	0.73065	0.91207	0.30117
C110	0.8043	0.75565	0.24139
C111	0.58795	0.47254	0.04213
C112	0.55676	0.33837	0.94562
C113	0.99552	0.28005	0.94261
C114	1.00995	0.24592	0.89495
C115	0.58128	0.26939	0.84902
C116	0.65386	0.32964	0.85051
C117	0.64156	0.36388	0.89861
N118	0.61322	0.22903	0.80431
C119	0.49454	-0.00151	0.47118
C120	0.52169	-0.00107	0.35796
C121	0.94826	0.05152	0.44095
C122	0.87748	0.10097	0.46938
C123	0.90863	0.05077	0.61402
C124	0.59358	0.8979	0.65382
C125	0.64639	0.83981	0.63544
C126	0.70423	0.79542	0.67216
C127	0.70963	0.80853	0.72786
C128	0.65767	0.8662	0.7463
C129	0.60091	0.91005	0.70987
C130	0.76617	0.76248	0.76737
C131	0.94995	0.46498	0.957
C132	0.60908	0.3352	0.04563

C133	0.69249	0.27885	0.03951
C134	0.73429	0.24334	0.08436
C135	0.69555	0.26334	0.13698
C136	0.61333	0.31971	0.14454
C137	0.56957	0.3551	0.09905
N138	0.74224	0.22456	0.18116
C139	0.52916	0.43565	0.99929
N140	0.56197	0.37005	0.99745
C141	0.56733	0.94756	0.44357
C142	0.62725	0.89822	0.47405
C143	0.52889	0.94707	0.61691
C144	0.83882	0.10181	0.64768
C145	0.8627	0.16427	0.63239
C146	0.81059	0.21115	0.66789
C147	0.73369	0.196	0.71897
C148	0.70841	0.13386	0.73401
C149	0.76085	0.08748	0.69891
C150	0.69325	0.24431	0.75864
C151	0.9184	0.52805	0.95781
C152	0.94444	0.6623	0.05436
C153	0.52039	0.721	0.05722
C154	0.50728	0.7552	0.10486
C155	0.91727	0.73113	0.15099
C156	0.84698	0.67055	0.14948
C157	0.8606	0.63639	0.10137
N158	0.88377	0.77131	0.19572
C159	0.99352	0.9999	0.52895
C160	0.96636	0.99958	0.64217
H161	0.17112	0.85756	0.55393
H162	0.33438	0.16766	0.41017
H163	0.22929	0.24806	0.34366
H164	0.31441	0.11919	0.20871
H165	0.41701	0.03972	0.27499
H166	0.12673	0.27394	0.24725

H167	0.11172	0.56654	0.07809
H168	0.27408	0.73759	1.00348
H169	0.19926	0.80249	0.92183
H170	0.41774	0.66335	0.81247
H171	0.49884	0.59941	0.89496
H172	0.28844	0.13938	0.54779
H173	0.07501	0.8233	0.40923
H174	0.16917	0.73834	0.34485
H175	0.35558	0.87902	0.22471
H176	0.25928	0.96305	0.28839
H177	0.2678	0.70546	0.25092
H178	0.16977	0.45201	0.07649
H179	0.40543	0.25882	0.98013
H180	0.43083	0.19634	0.89365
H181	0.23602	0.35288	0.81244
H182	0.21137	0.41482	0.8997
H183	0.04428	0.00221	0.31138
H184	0.31142	0.13989	0.44626
H185	0.1471	0.82989	0.59003
H186	0.24905	0.74895	0.65658
H187	0.16892	0.87828	0.79148
H188	0.06942	0.95828	0.72516
H189	0.3198	0.71675	0.75246
H190	0.39325	0.43496	0.92185
H191	0.24256	0.26465	-0.00366
H192	0.31989	0.20015	0.07796
H193	0.096	0.33816	0.18738
H194	0.01242	0.40167	0.10492
H195	0.19411	0.85796	0.45241
H196	0.40624	0.17459	0.59098
H197	0.30879	0.25906	0.65539
H198	0.12771	0.11755	0.77549
H199	0.22727	0.03403	0.71177
H200	0.17808	0.29172	0.74582

H201	0.33964	0.54982	0.9234
H202	0.09278	0.74254	0.01988
H203	0.06496	0.80486	0.10639
H204	0.27722	0.64931	0.18741
H205	0.29948	0.58722	0.10018
H206	0.44385	0.99593	0.68875
H207	0.66471	0.85681	0.554
H208	0.8529	0.17011	0.40997
H209	0.75095	0.25105	0.34342
H210	0.83108	0.12172	0.20852
H211	0.93058	0.04172	0.27484
H212	0.6802	0.28325	0.24754
H213	0.60675	0.56504	0.07815
H214	0.77231	0.7374	1.00349
H215	0.69498	0.8019	0.92188
H216	0.91887	0.6639	0.81246
H217	1.00245	0.60038	0.89492
H218	0.80589	0.14204	0.54759
H219	0.56989	0.82211	0.40929
H220	0.66734	0.73764	0.34487
H221	0.84841	0.87915	0.22478
H222	0.74885	0.96267	0.2885
H223	0.76726	0.70526	0.25093
H224	0.66036	0.45018	0.0766
H225	0.92209	0.25952	0.97996
H226	0.94991	0.1972	0.89345
H227	0.72278	0.35069	0.81259
H228	0.70052	0.41278	0.89982
H229	0.53228	0.00077	0.31151
H230	0.82888	0.14244	0.44607
H231	0.64175	0.82904	0.59009
H232	0.74684	0.74864	0.6566
H233	0.66171	0.87751	0.79156
H234	0.55911	0.95698	0.72527

H235	0.81885	0.71679	0.75247
H236	0.90315	0.43551	0.92175
H237	0.72592	0.26241	-0.00348
H238	0.80074	0.19751	0.07817
H239	0.58226	0.33665	0.18753
H240	0.50116	0.40059	0.10504
H241	0.68769	0.85732	0.45248
H242	0.92499	0.1767	0.59077
H243	0.83083	0.26166	0.65515
H244	0.64442	0.12098	0.77529
H245	0.74072	0.03695	0.71161
H246	0.7322	0.29454	0.74908
H247	0.8451	0.55005	0.92334
H248	0.59457	0.74118	0.01987
H249	0.56917	0.80366	0.10635
H250	0.77885	0.64918	0.1874
H251	0.8035	0.58723	0.10013
H252	0.95572	0.99779	0.68862

Table S2. Unit cell parameters of PyT-2 with AA stacking structure.

Section S3 SEM analysis

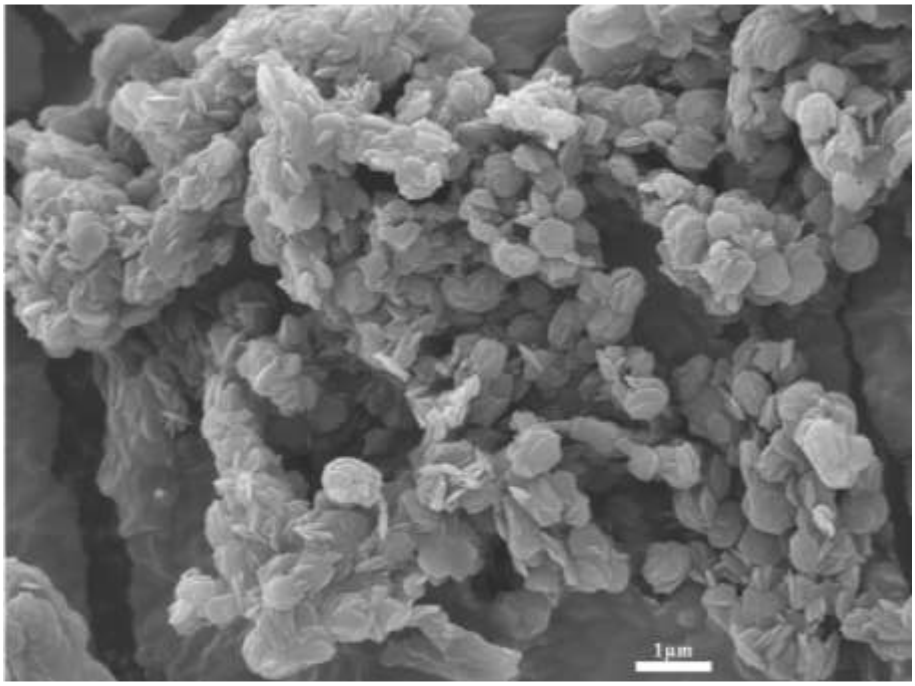

Figure S2. SEM image of PyT-1.

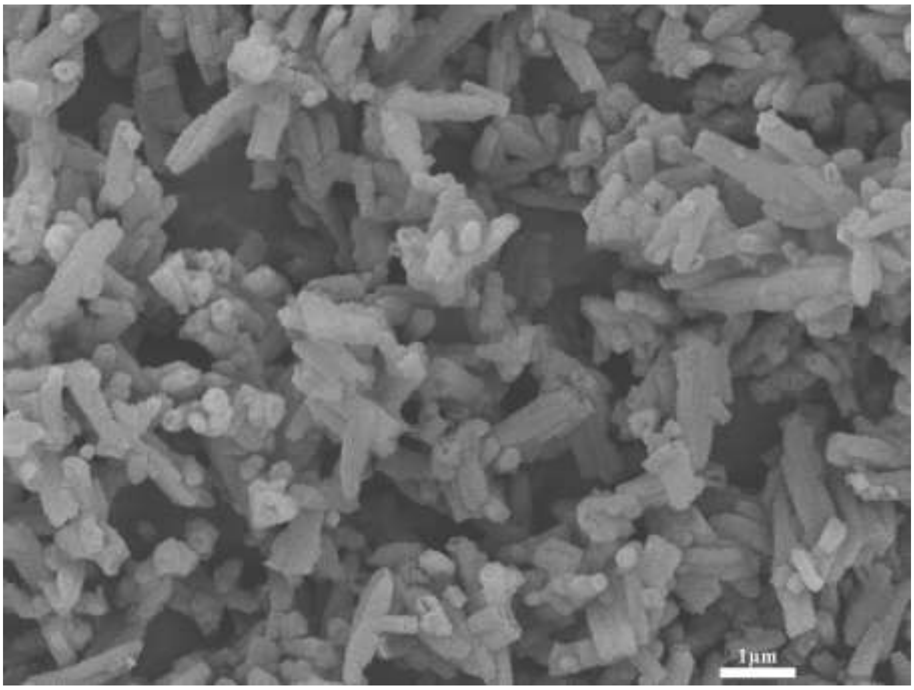

Figure S3. SEM image of PyT-2.

Section S4 Thermogravimetric analysis and structural stability

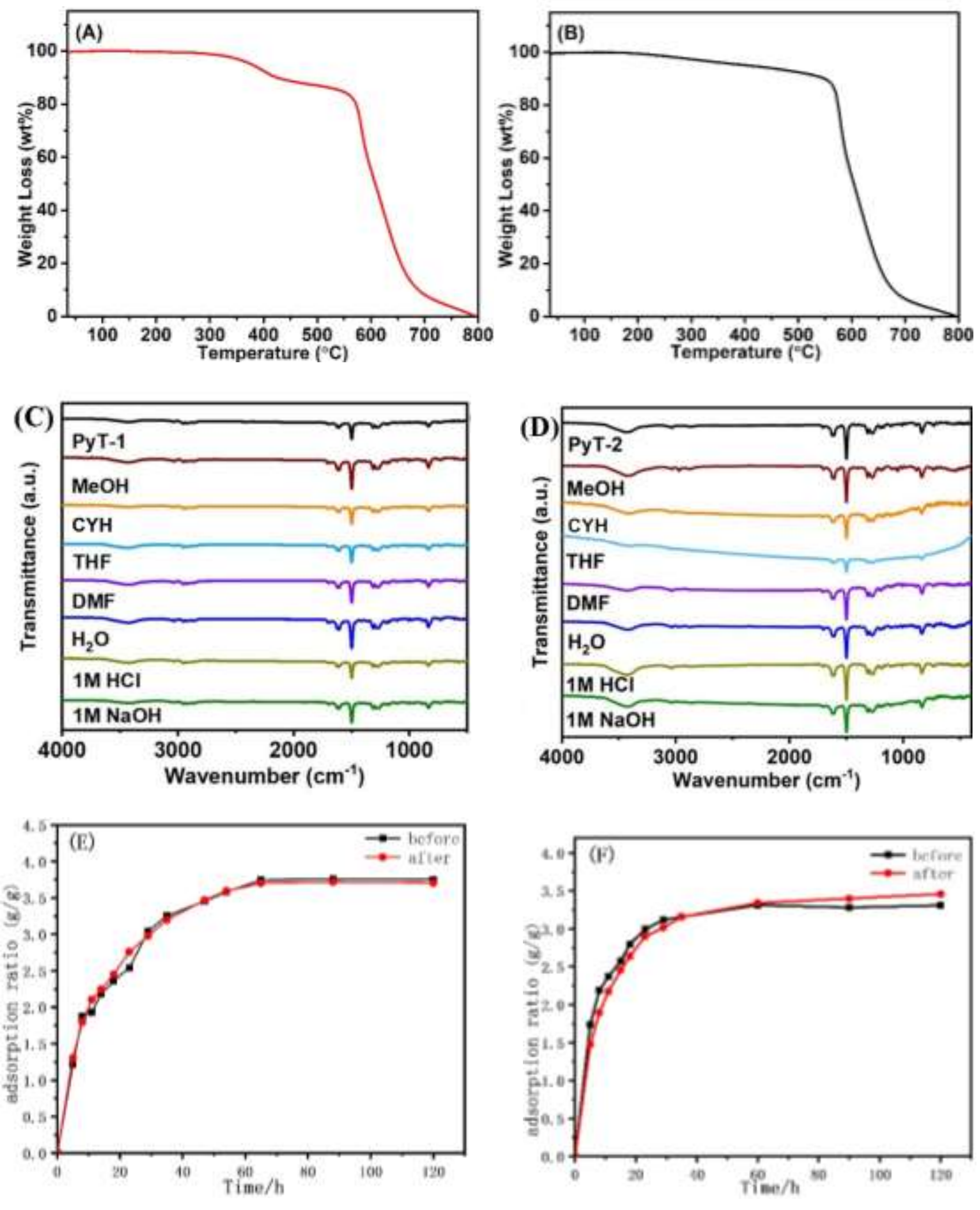

Figure S4. TGA curves of PyT-1 (A) and PyT-2 (B), FTIR spectra in various solvents of PyT-1 (C) and PyT-2 (D) and iodine vapor adsorption capacities of PyT-1(E), PyT-2(F) before and after being immersed in n-hexane.**Section**

S5 Iodine vapor adsorption experiment

The experiments were carried out as follows: COFs (5mg) in bottle were exposed in a sealed device (10ml) filling with excess iodine. The bottles were removed periodically, cooled to room temperature, and removed to measure the overall difference in the mass of the vials before and after. Adsorption stability was measured by weighing the daily mass change of the adsorbed material over a period of seven days at room temperature and pressure. The recyclability of COFs for capturing of iodine vapor was carried out 5 cycles after the COFs were rinsed with ethanol to release iodine.

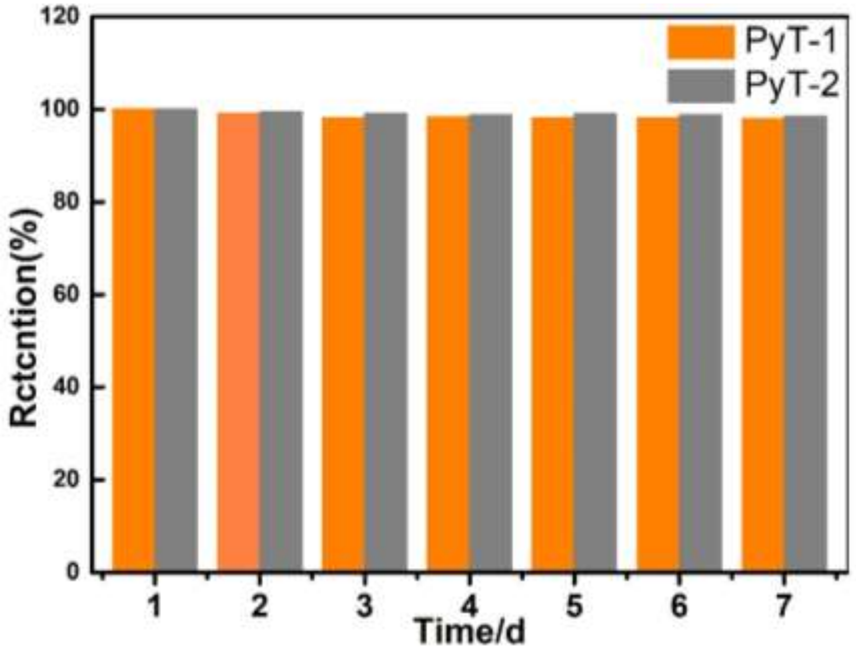

Figure S5. iodine retention of PyT-1, PyT-2 exposure to air at 25 °C and ambient pressure after iodine capture.

Section S6 Iodine solution adsorption experiment

Iodine containing solutions at concentrations of 400 mg L^{-1} and 200 mg L^{-1} were prepared, and 20 ml of each solution was added to the volumetric flask with 2 mg COF powder in it. The amount of iodine absorbed by COF can also be calculated by the equation (1). The pseudo-first-order and pseudo-second-order kinetic models were used to study the adsorption kinetics of iodine solution according to equation (2) and equation (3), respectively.

$$q_t = q_e\ (\ 1 - exp\ (-k1t)\ )\ (2)$$

$$q_t = (k_2 q_e^2 t) / (\ 1 + k_2 qet\ )\ (3)$$

where q_e (g g^{-1}) and q_t (g g^{-1}) are the adsorption value at equilibrium and time t (min), respectively. k_1 (min^{-1}) and k_2 (min^{-1}) are the rate constant of pseudo-first-order and pseudo-second-order adsorption, respectively (details are given in Table S4).

Porous material	I_2 solution concentration (mg L^{-1})	I_2 solution uptake (mg g^{-1})	Ref.
NOP-54	254	22.61	[1]
TFB-BD COF	500	99.90	[2]
Tfp-DB COF	500	99.9	[3]
NT-POP@800-1	4000	396.0	[4]
PAN-1	2540	333.3	[5]
P-DPDA	400	450	[6]
	200	150	
CACY	400	245	[7]
POP-T	500	902.8	[8]
P-COF	300	1295.2	[9]
PyT-1	400	635	This work
	200	365	
PyT-2	400	445	This work
	200	315	

Table S3. Summary of representatively reported adsorbents with our work for iodine solution adsorption under ambient pressure.

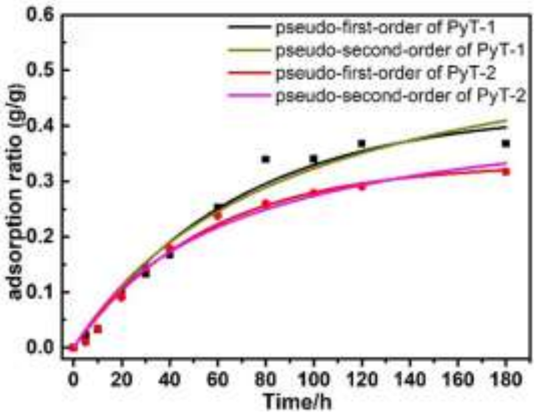

Figure S6. Iodine adsorption capacities of PyT-1, PyT-2 in n-hexane with iodine concentration of 200 mg L^{-1} (B).

	$c(I_2)$	q_e(exp) (mg g^{-1})	Pseudo-first-order kinetic model			Pseudo-second-order kinetic model		
			K_1(min^{-1})	q_e (mg g^{-1})	R^2	K_1(min^{-1})	q_e (mg g^{-1})	R^2
PyT-1	400 mg g^{-1}	635	0.029	636	0.998	0.014	780	0.995
PyT-2		445	0.031	442	0.993	0.005	542	0.991
PyT-1	200 mg g^{-1}	365	0.015	428	0.975	0.003	613	0.964
PyT-2		315	0.018	332	0.990	0.001	453	0.982

Table S4. Kinetic parameters for the volatile iodine adsorption onto PyT-1 and PyT-2.

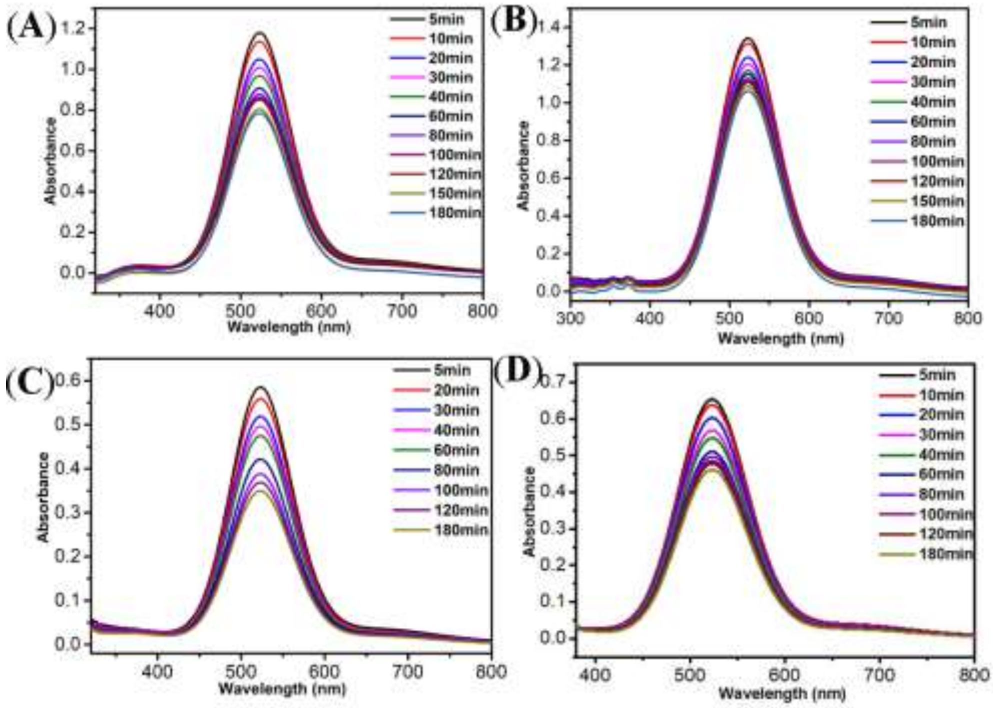

Figure S7. (A) UV-vis spectra for PyT-1 in 400 mg L-1 of iodine in n-hexane solution at different times (B) UV-vis spectra for PyT-2 in 400 mg L-1 of iodine in n-hexane solution at different times (C) UV-vis spectra for PyT-1 in 200 mg L-1 of iodine in n-hexane solution at different times (D) UV-vis spectra for PyT-2 in 200 mg L-1 of iodine in n-hexane solution at different times.

References:

[1] Chen, D.; Fu, Y.; Yu, W, et al. Versatile Adamantane-based Porous Polymers with Enhanced Microporosity for Efficient CO 2 Capture and Iodine Removal[J]. Chemical Engineering Journal, 2018: 900-906.

[2] Song, S.; Shi, Y.; Liu, N, et al. Theoretical Screening and Experimental Synthesis of Ultrahigh-Iodine Capture Covalent Organic Frameworks[J]. ACS Appl Mater Interfaces, 2021, 13(8): 10513-10523.

[3] Sanansong, Yueshi, Ningliu, et al. C[double bond, length as m-dash]N linked covalent organic framework for the efficient adsorption of iodine in vapor and solution[J]. RSC Advances, 11.

[4] Yao, C.; Li, G.; Wang, J, et al. Template-free synthesis of porous carbon from triazine based polymers and their use in iodine adsorption and CO 2 capture[J]. Scientific Reports, 2018, 8(1): 1867.

[5] Ren, Y. Y.; Zhang, W. Y.; Zhu, Y. L, et al. Nitrogen-rich porous polyaminal network as a platform for iodine adsorption through physical and chemical interaction[J]. Journal of Applied Polymer Science, 2018, 135(15).

[6] Wang, J.; Wang, C. C.; Wang, H. C, et al. Synthesis of N-containing porous aromatic frameworks via Scholl reaction for reversible iodine capture[J]. Microporous and Mesoporous Materials, 2021, 310: 110596.

[7] Jz, A.; Sf, B.; Yw, A. et al. The adsorbent for efficient iodine capture based on citrazinic acid and cytosine: experimental synthesis with a simple way and property analysis with electronic structure calculations - ScienceDirect[J], 2021.

[8] Tian, P.; Ai, Z.; Hu, H, et al. Synthesis of Electron-Rich Porous Organic Polymers via Schiff-Base Chemistry for Efficient Iodine Capture[J]. Molecules, 2022, 27(16): 5161.

[9] Li, Y.; Li, X. G.; Li, J. F, et al. Phosphine-based covalent organic framework for highly efficient iodine capture[J]. Microporous and Mesoporous Materials, 2021, 325.
